# Evaluation of an online knowledge translation intervention to promote cancer risk reduction behaviours: findings from a randomized controlled trial

**DOI:** 10.1186/s12885-019-6361-2

**Published:** 2019-11-21

**Authors:** Sarah E. Neil-Sztramko, Emily Belita, Anthony J. Levinson, Jennifer Boyko, Maureen Dobbins

**Affiliations:** 1 McMaster University, 175 Longwood Rd South, Suite 210a, Hamilton, ON L8P 3Y2 Canada; 2 The National Collaborating Centre for Methods and Tools, McMaster University, Hamilton, ON Canada

**Keywords:** Knowledge translation, Cancer prevention, Physical activity, Diet, Alcohol, Tobacco

## Abstract

**Background:**

Many cancers are preventable through lifestyle modification; however, few adults engage in behaviors that are in line with cancer prevention guidelines. This may be partly due to the mixed messages on effective cancer prevention strategies in popular media. The goal of the McMaster Optimal Aging Portal (the Portal) is to increase access to trustworthy health information. The purpose of this study was to explore if and how knowledge translation strategies to disseminate cancer prevention evidence using the Portal influence participants’ knowledge, intentions and health behaviors related to cancer risk.

**Methods:**

Adults ≥40 years old, with no cancer history were randomized to a 12-week intervention (weekly emails and social media posts) or control group. Quantitative data on knowledge, intentions and behaviors (physical activity, diet, alcohol consumption and use of tobacco products) were collected at baseline, end of study and 3 months later. Participant engagement was assessed using Google Analytics, and participant satisfaction through open-ended survey questions and semi-structured interviews.

**Results:**

Participants (*n* = 557, mean age 64.9) were predominantly retired (72%) females (81%). Knowledge of cancer prevention guidelines was higher in the intervention group at end of study only (+ 0.3, *p* = 0.01). Intentions to follow cancer prevention guidelines increased in both groups, with no between-group differences. Intervention participants reported greater light-intensity physical activity at end of study (+ 0.7 vs. 0.1, *p* = 0.03), and reduced alcohol intake at follow u (− 0.2 vs. + 0.3, *p* < 0.05), but no other between-group differences were found. Overall satisfaction with the Portal and intervention materials was high.

**Conclusions:**

Dissemination of evidence-based cancer prevention information through the Portal results in small increases in knowledge of risk-reduction strategies and with little to no impact on self-reported health behaviours, except in particular groups. Further tailoring of knowledge translation strategies may be needed to see more meaningful change in knowledge and health behaviours.

**Trial registration:**

ClinicalTrials.gov NCT03186703, June 14, 2017.

## Background

In Canada, an estimated one-third to one-half of all cancers are preventable through lifestyle modification such as smoking cessation, increasing physical activity, healthy eating, and reducing alcohol intake [1, 2]. Despite this, few Canadians engage in behaviours that are in line with evidence-based cancer prevention guidelines; 20% of Canadian adults smoke [3], 85% do not meet physical activity guidelines [4], 77% eat less than five servings of fruits and vegetables per day [5], and 20% of men and 8% of women consume more than two alcoholic drinks per day [6].

Although people generally understand that they should eat better, exercise more, drink less and not smoke, there is a lack of awareness of the link between these lifestyle behaviours and cancer risk. In a survey of Canadian adults, while 90% were aware of the link between smoking and cancer, knowledge was much lower for the link between cancer risk and diet (52%), alcohol intake (33%), being overweight (31%) and physical inactivity (28%) [7]. Similar results have been found in other countries. In a national survey of US adults, only 44% believed that individual behaviours contributed substantially to the risk of developing cancer [8], and a UK survey found only a small proportion of adults were aware that poor diet (32%), physical inactivity (22%) and frequent alcohol intake (33%) contributed to cancer risk [9]. While the acquisition of knowledge on cancer prevention is only one component of the process of behaviour change and reducing cancer risk, an effective and scalable knowledge translation (KT) strategy that can increase public knowledge of evidence-based cancer prevention recommendations may be an important step in this pathway.

Increasingly, many people turn to the internet and social media as a source of health information [10–13]. A study of online searchers using Google AdWords found that over an 11-month period there were over 117 million unique searches in Canada alone related to cancer prevention (physical activity/exercise, healthy eating, weight loss and quitting smoking) [11]. Unfortunately, much of the health information available online is not based on scientific evidence [14, 15]. Members of the public may not have the knowledge, skills or time to sift through and identify credible messages [16–18] and may be acting on recommendations which are unlikely to improve health. A National Cancer Institute survey found that almost half of Americans reported seeking out information about cancer online; of these, 58% of reported concerns about the quality of information [19], 48% found the search to require a lot of effort and 41% found it to be frustrating. Importantly, those with negative experiences with the process of searching for cancer information online were more likely to have inaccurate knowledge and beliefs about what can be done to prevent cancer [19].

The *McMaster Optimal Aging Portal* (the Portal) was launched in English in 2014, and French in 2017, as an online repository of evidence-based information to increase public access to trustworthy health information related to healthy aging [20]. Content (blog posts, evidence summaries and web-resource ratings) are developed and maintained through a team at McMaster University, and aim to provide easy-to-read, ‘bottom line’ messages appropriate for all audiences, with or without previous medical or scientific knowledge and training (see previous publications for a description of Portal development and content [21–24]). In addition to being a source for accessible and trustworthy cancer prevention information for Canadian adults, it has potential to be particularly helpful for underserved populations and those in rural and remote locations who experience barriers to accessing health information through a healthcare provider, for example.

Evidence from recent systematic reviews suggests that websites and social media have the potential to improve health behaviours, self-efficacy [25] and health outcomes [26], including those related to cancer prevention [27]. For example, access to credible and reliable web information is associated with compliance to evidence-based recommendations for colorectal cancer screening [28]. However, it is not known if access to high-quality information about cancer prevention results in behaviour changes such as smoking cessation, increased physical activity, healthy eating, and reduced alcohol consumption. The purpose of this study is to understand if and how KT strategies used to disseminate information about cancer prevention through the Portal impact knowledge, intentions and health behaviours. A secondary aim was to compare outcomes in rural Canadians to those who live in urban areas.

## Methods

Using a sequential explanatory mixed-methods design [29], we evaluated the Portal’s existing KT strategies to disseminate research on cancer prevention – specifically related to smoking, physical activity, healthy eating, and alcohol intake – to study participants. A two-arm randomized controlled trial (RCT) was conducted, followed by a qualitative process study to explore the findings from the RCT in greater depth. This approach, rather than a simple RCT, was selected to allow for a deeper analysis of not only the quantitative outcomes of interest, but also to gain greater understanding of the KT process. Ethical approval was provided by the Hamilton Integrated Research Ethics board (ID: 3285) and all participants provided written, informed consent.

### Participants

Eligible participants were English-speaking adults, ≥40 years old who had never been diagnosed with cancer. Participants were recruited from November 2017 to January 2018 through a link to study information on the Portal’s email subscription list and social media posts, and through partner organizations including the McMaster Institute for Research on Aging (and its partners), MedicAlert® Canada, and the Canadian Association of Retired Teachers. Through the online link, participants were provided with the study consent form and baseline questionnaire. Using a conservative estimate of a small effect size (0.16, from a meta-analysis of internet health behaviour change interventions [30]), with a power of 0.80 and alpha of 0.05, we required a total of 388 participants in the study. To allow for a 25% drop out rate, we aimed to recruit 485 individuals.

### Study protocol

Following baseline data collection, participants were stratified by previous Portal use and urban/rural location (defined using postal code) and randomized to a 12-week KT intervention or control group in a 1:1 ratio. A computer-generated random numbers sequence was used by an individual not involved with recruitment or data collection. The sequence was generated, and randomization was completed after all baseline data collection were complete, thus allocation was concealed from study participants and research personnel. Due to the nature of the study, participants were not blinded.

**Fig. 1:**
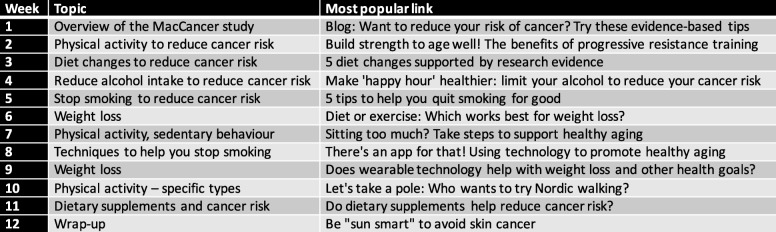
Weekly intervention topics

Intervention group participants received targeted weekly email alerts that included links to blog posts and evidence summaries relevant to cancer prevention on the Portal (Fig. 1). In the first week of the study, participants were invited via email to follow a Twitter and Facebook feed using a study-specific hashtag (#MacCancer), and a cancer prevention ‘Browse’ page. As the Portal is publicly available, control group participants were able to access the Portal if they wished, but were not directed to the cancer prevention content, and did not receive targeted KT strategies. They were asked to continue their normal lifestyle throughout the study and told all study-related information would be shared at the end of the follow-up period.

The KT intervention was informed by the Theory of Planned Behaviour (TPB). The TPB [31] has been used and tested extensively with respect to health behaviours including physical activity [32, 33] smoking [34], healthy eating [35], and alcohol consumption [36]. The TPB suggests that intention to engage in a particular behaviour is an immediate precursor of the behaviour, and that intention is based on attitude toward the behaviour, subjective norms, and perceived behavioural control [37, 38]. Through the KT intervention, we aimed to modify individuals’ attitudinal beliefs through the provision of evidence-based information about cancer risk reduction strategies. The content provided was targeted towards our population of middle-aged and older adults, and included actionable messages within the content, to act on normative and control beliefs.

### Outcome measures

Quantitative data were collected via web-administered questionnaires with established reliability and validity at baseline, the end of the 12-week intervention, and 3 months post-intervention. ***Knowledge*** of cancer prevention recommendations and guidelines were collected using true/false questions about cancer prevention recommendations related to each health behaviour. For each, participants were classified as correct or incorrect, and the total was summed to create a total knowledge score. ***Intentions*** to engage in health behaviours in line with guidelines were assessed using a 7-point Likert scale. ***Smoking status*** was assessed using questions from the Tobacco Questions for Surveys tool from the World Health Organization [39]. ***Physical activity*** was measured using the Godin Leisure Time Exercise Questionnaire, which allows calculation of an overall physical activity score, with > 24 points being classified as ‘active’ [40]. ***Dietary intake*** was assessed using the 16-item Dietary Screener Questionnaire (DSQ) to assess frequency of consumption of food and drink related to cancer risk, specifically fruit and vegetable, whole grains and fiber intake [41]. Current ***alcohol intake*** was reported using a seven-day recall, which has been found to provide values comparable to summary measures of alcohol use [42–44]. Self-rated health was measured on a 5-point Likert Scale [45], and eHealth literacy was measured using the eHealth Literacy Scale [46].

Data related to engagement with the KT strategies were collected during and after the intervention via Google analytics for the intervention group only in order to more fully understand how engagement with materials may impact changes in knowledge, intentions and health behaviours. The following metrics were used: number of unique users; bounce rate (the proportion of individuals who only viewed one page per session); number, frequency and length of sessions; number of page views; average time on pages, and pageviews by topic. Self-reported use of email alerts, social media posts, and Portal browse page was collected via end of study questionnaires in both groups.

### Qualitative data collection

Qualitative data were collected from participants in two ways. First through open-ended questions in end of study and follow-up questionnaires (all participants). Second, via semi-structured interviews with a purposeful subsample of interested participants (*n* = 35). A trained interviewer who had no previous involvement with the study conducted interviews by phone. Participants were included from both intervention and control groups, and our sampling aimed for a balance of males and females, urban and rural adults, and those who had and had not used the Portal previously. Qualitative questions explored satisfaction with the KT strategies and information received (intervention group only), satisfaction with the Portal in general (both groups), and whether the Portal/KT strategies are a feasible way to disseminate information and how attitudes, beliefs and behaviours changed during the study. All interviews were recorded and transcribed verbatim.

### Data analysis

Between-group differences in outcome measures at end of study and post-intervention follow-up were analyzed using an intention-to-treat analysis using a two-way mixed-effects generalized linear model, with the interaction of intervention group by time as the main feature of interest at each time point, with baseline values included in the model. Subgroup analyses were specified a priori to examine differences in outcomes for urban vs. rural participants, by baseline self-rated health, and by those who had and had not used the Portal previously. Engagement and satisfaction were summarized using descriptive statistics. Comparison between groups was done using t-tests for continuous data or chi-square tests for categorical variables.

Qualitative data from interview transcripts were entered into NVivo 12 software for data storage, indexing, searching, and coding (QSR International, Melbourne AUS). An inductive approach was used to code and analyze the qualitative data. Three members of the study team (SNS, EB and a research assistant) analyzed a subset of 10 transcripts independently using open coding and then met to come to agreement on a coding scheme of lower and higher order categories (e.g., intervention process, changes in behaviour). The remaining transcripts were then divided amongst the team for coding using the agreed upon scheme. The team met a second time to finalize and agree upon coding and interpretation of results.

## Results

**Fig. 2:**
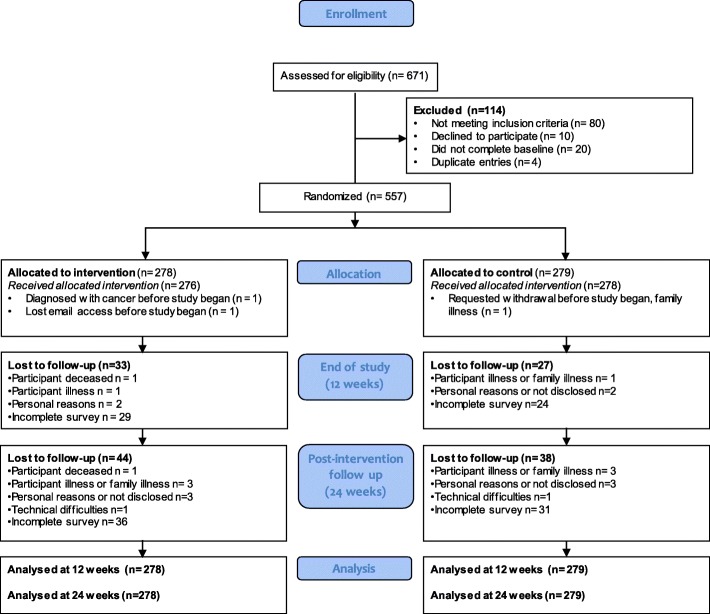
Participant flow through study

Of 671 individuals who responded to online recruitment, 557 eligible participants completed the baseline questionnaire and were randomized to the intervention (*n* = 278) or control group (*n* = 279) (Fig. 2). Retention was high, with 88.3 and 84.2% of participants completing end of study and follow-up questionnaires respectively. Participants who failed to complete the end of study questionnaire were eligible and invited to complete the follow-up. There were no differences in loss to follow-up between groups. Participants who did not complete the end of study questionnaire were more likely to have not previously used the Portal (68.3% vs. 51.5%, *p* = 0.01), have lower baseline self-rated health (3.6 vs. 3.9 on a 5-point Likert scale, *p* = 0.02), and have lower baseline physical activity (28.7 vs. 35.3 points, *p* = 0.03). Participants who did not complete the follow-up questionnaire were more likely to have never used the Portal (58.8 vs. 52.4, *p* = 0.02), and have lower baseline physical activity (26.4 vs. 36.0 points, *p* < 0.001). No other descriptive characteristics were associated with loss to follow-up.

There were no baseline differences in demographic characteristics between the intervention and control groups (Table 1). Participants were predominantly older (65.2 ± 8.0 years), retired (71.6%) female (80.3%), and well-educated (94.1% had post-secondary education, and one-third had a post-graduate degree). Despite 51.4% reporting at least one chronic condition, 71.1% rated their health as ‘excellent’ or ‘very good’. Half of participants had never used the Portal before, and one-quarter were regular users.

**Table 1:** Participant characteristics

	Total	Intervention	Control	*p*
	*n* = 557	*n* = 278	*n* = 279	
	Mean ± SD			
Age	65.2 ± 8.0	65.4 ± 7.9	64.9 ± 8.2	0.51
	N (%)			
Female	447 (80.3)	221 (79.5)	226 (81.0)	0.73
Education (%)				
Secondary school or less	33 (5.9)	19 (6.9)	14 (5.0)	0.43
Post-secondary diploma	102 (18.3)	49 (17.6)	53 (19.1)	
Bachelor’s degree	235 (42.3)	121 (43.5)	114 (41.0)	
Post-graduate degree	186 (33.5)	89 (32.0)	97 (34.9)	
Employment status (%)				
Full-time	92 (16.5)	51 (18.4)	41 (14.7)	0.75
Part-time	59 (10.6)	27 (9.7)	32 (11.5)	
Homemaker	4 (0.7)	2 (0.7)	2 (0.7)	
Retired	398 (71.6)	196 (70.8)	202 (72.4)	
Long-term disability	3 (0.5)	1 (0.4)	2 (0.7)	
Geographic location (%)				
Urban	248 (44.5)	119 (42.8)	129 (46.2)	0.63
Suburban	207 (37.2)	107 (38.5)	100 (35.8)	
Rural/Remote	102 (18.3)	52 (18.7)	50 (18.0)	
Chronic disease	285 (51.4)	144 (52.0)	141 (50.9)	0.87
Self-rated health ‘Excellent’ or ‘Very Good’	396 (71.1)	203 (73.0)	193 (69.1)	0.35
Previously used the McMaster Optimal Aging Portal (%)				
No	297 (53.4)	148 (53.2)	149 (53.6)	0.79
Yes, regular user	140 (25.2)	73 (26.3)	67 (24.1)	
Yes, occasionally	119 (21.4)	57 (20.5)	62 (22.3)	

### Changes in knowledge, intentions and health behaviours

There were no differences between groups at baseline in knowledge, intentions or health behaviours (Table 2). Only three participants in the study reported being current smokers (data not shown), therefore changes in knowledge, intentions and smoking behaviours were not analyzed. Baseline knowledge of cancer prevention guidelines was high (mean 4.6 out of 5 guidelines correctly identified). Knowledge was highest for fruit and vegetable intake (98%) and lowest for alcohol (80.1%). At end of study and follow-up, total knowledge score was significantly higher in the intervention vs. control group. At end of study, intervention participants were significantly more likely than controls to identify physical activity and alcohol guidelines (OR: 5.57, 95% CI: 1.20, 25.79 and OR: 2.05, 95% CI: 1.02, 41.2 respectively), and at follow-up were more likely to identify red meat and fiber intake guidelines (OR: 3.00, 95% CI: 1.03, 8.71).

**Table 2:** Quantitative outcomes at baseline, end of study and follow-up amongst intervention and control participants

	Baseline			End of Study			Follow-up		
	Intervention	Control	*p*	Intervention	Control	*p*	Intervention	Control	*p*
Total Knowledge (Correct answers)	4.6 (4.5, 4.7)	4.6 (4.5, 4.7)	0.56	**+ 0.3 (0.2, 0.4)**	**+ 0.1 (0.0, 0.2)**	**0.01**	+ 0.3 (0.2, 0.4)	+ 0.2 (0.1, 0.3)	0.09
Physical activity ^a^	91.7 (87.9, 94.4)	90.7 (86.7, 93.6)	0.55	**5.57 (1.20, 25.79)**	**REF**	**0.03**	1.58 (0.37, 6.81)	REF	0.54
Alcohol ^a^	80.2 (75.1, 84.5)	81.4 (76.4, 85.5)	0.68	**2.05 (1.02, 4.12)**	**REF**	**0.04**	1.29 (0.60, 2.76)	REF	0.52
Fruit & Vegetable ^a^	98.2 (95.9, 99.2)	97.5 (94.9, 98.8)	0.46	1.46 (0.24, 8.95)	REF	0.68	1.91 (0.17, 21.5)	REF	0.60
Red meat/fiber ^a^	88.9 (84.6, 92.0)	91.8 (87.9, 94.5)	0.17	1.89 (0.82, 4.33)	REF	0.13	**3.00 (1.03, 8.71)**	**REF**	**0.04**
Intentions (7-point Likert scale)	5.8 (5.7, 5.9)	5.7 (5.6, 5.9)	0.46	+ 0.3 (0.1, 0.4)	+ 0.3 (0.1, 0.4)	0.51	+ 0.3 (0.2, 0.4)	+ 0.3 (0.2, 0.4)	0.72
Physical activity	5.5 (5.3, 5.7)	5.4 (5.2, 5.6)	0.52	+ 0.4 (0.2, 0.6)	+ 0.3 (0.2, 0.5)	0.41	+ 0.3 (0.1, 0.5)	+ 0.4 (0.2, 0.6)	0.81
Alcohol	5.9 (5.7, 6.1)	5.9 (5.7, 6.1)	0.89	+ 0.2 (−0.002, 0.4)	+ 0.2 (0.02, 0.5)	0.99	+ 0.2 (−0.02, 0.4)	+ 0.2 (− 0.01, 0.4)	0.95
Fruit & Vegetable	5.7 (5.5, 5.9)	5.7 (5.6, 5.9)	0.92	+ 0.3 (0.1, 0.4)	+ 0.2 (0.1, 0.4)	0.90	+ 0.4 (0.2, 0.6)	+ 0.3 (0.2, 0.5)	0.72
Red meat/fiber	6.0 (5.9, 6.2)	5.9 (5.8, 6.1)	0.42	+ 0.2 (0.002, 0.3)	+ 0.2 (−0.002, 0.3)	0.42	+ 0.1 (− 0.05, 0.3)	+ 0.2 (0.04, 0.4)	0.91
Physical Activity									
Total PA score	35.3 (32.5, 38.1)	33.8 (31.0, 36.6)	0.46	+ 4.6 (−0.1, 8.1)	+ 2.1 (− 0.2, 4.5)	0.14	+ 5.5 (3.0, 7.9)	+ 4.1 (1.7, 6.5)	0.43
Strenuous PA (bouts/week)	1.1 (0.9, 1.3)	1.0 (0.8, 1.2)	0.49	+ 0.1 (−0.1, 0.3)	−0.0 (− 0.2, 0.2)	0.58	0.0 (− 0.2, 0.2)	+ 0.1 (− 0.1, 0.3)	0.61
Moderate PA (bouts/week)	2.9 (2.6, 3.2)	2.7 (2.4, 3.0)	0.55	+ 0.4 (0.1, 0.7)	+ 0.4 (0.1, 0.7)	0.90	+ 0.6 (0.3, 0.9)	+ 0.4 (0.1, 0.7)	0.38
Light PA (bouts/week)	3.6 (3.3, 4.0)	3.6 (3.3, 4.0)	0.99	**+ 0.7 (0.3, 1.1)**	**+ 0.1 (−0.3, 0.5)**	**0.03**	+ 0.8 (0.5, 1.2)	+ 0.6 (0.2, 0.9)	0.28
Alcohol intake (servings/week)									
Total	5.2 (4.5, 6.0)	6.0 (5.2, 6.8)	0.18	−0.4 (− 0.8, 0.1)	− 0.5 (− 0.9, 0)	0.80	− 0.1 (− 0.6, 0.3)	− 0.1 (− 0.6, 0.3)	0.99
Beer	1.1 (0.7, 1.5)	1.4 (1.0, 1.9)	0.26	0.0 (− 0.2, 0.3)	0.0 (− 0.2, 0.3)	0.98	+ 0.5 (0.2, 0.7)	+ 0.5 (0.2, 0.7)	0.98
Wine	4.4 (3.8, 5.0)	4.6 (4.0, 5.2)	0.75	−0.3 (− 0.6, 0.0)	− 0.6 (−1.0, − 0.3)	0.22	−0.3 (− 0.6, 0.1)	−0.7 (− 1.1, − 0.4)	0.08
Liquor	1.2 (0.7, 1.6)	1.3 (0.8, 1.7)	0.66	−0.2 (− 0.5, 0.2)	+ 0.3 (− 0.0, 0.7)	0.07	**−0.2 (− 0.9, 0.4)**	**0.3 (− 0.0, 0.7)**	**< 0.05**
Diet									
Fruit/veg (servings per day)	3.0 (2.9, 3.1)	2.9 (2.8, 3.0)	0.17	+ 0.1 (0.01, 0.2)	+ 0.2 (0.1, 0.2)	0.16	+ 0.2 (0.1, 0.3)	+ 0.2 (0.1, 0.3)	0.80
Whole grains (servings per day)	0.9 (0.9, 1.0)	0.9 (0.9, 1.0)	0.80	0.0 (−0.0, 0.0)	0.0 (− 0.0, 0.0)	0.69	− 0.0 (− 0.1, 0.0)	−0.0 (− 0.1, 0.0)	0.53
Fiber (g/day)	18.2 (17.8, 18.5)	18.0 (17.7, 18.3)	0.48	+ 0.2 (−0.1, 0.4)	+ 0.3 (0.0, 0.6)	0.49	+ 0.2 (− 0.1, 0.5)	+ 0.3 (0.0, 0.5)	0.70
Self-rated Health	3.8 (3.7, 3.9)	3.8 (3.8, 3.9)	0.87	+ 0.02 (−0.1, 0.1)	−0.04 (− 0.1, 0.03)	0.20	0.03 (− 0.04, 0.1)	0.01 (− 0.1, 0.1)	0.70
E-health literacy score	30.4 (29.8, 31.1)	29.1 (28.5, 29.8)	**0.01**	**+ 1.4 (0.8, 1.9)**	**+ 0.6 (0.1, 1.1)**	**0.04**	+ 1.6 (1.0, 2.1)	+ 1.4 (0.8, 1.9)	0.65

*p*-value from generalized mixed model, group*time interaction at respective time points; ^a^end of study and follow-up data presented as odds ratio, comparing odds of correctly identifying guideline in intervention vs. control group

Bold indicates statistically significant within-group difference

Intentions to engage in recommended behaviours were also high at baseline in both groups, particularly for red meat and fiber intake (mean 6.0 on a 7-point Likert scale) and lowest for physical activity (5.5 on a 7-point Likert scale). There were no between-group differences in intentions at end of study or follow-up with respect to behavioural intentions.

At end of study, there was a significant between-group difference for number of bouts of light physical activity per week (+ 0.6, *p* = 0.03), eHealth literacy (+ 0.8 points, *p* = 0.04), and knowledge (+ 0.2, *p* = 0.01) favoring the intervention group. No between-group differences were found in total physical activity score, bouts of strenuous or moderate activity, self-rated health, or any measures of alcohol or dietary intake. At post-intervention follow-up, the only between-group difference was serving per week of liquor, favoring the intervention group (− 0.5, *p* < 0.05).

A secondary aim was to examine the effect of the intervention amongst rural Canadians. We hypothesized that rural Canadians who may have more limited access to health care providers may be more likely to benefit from the intervention. At end of study, there were no between-group differences in total physical activity for those who lived in urban/suburban settings (+ 3.3, *p* = 0.07) or rural settings (+ 1.8, *p* = 0.26). No between-group differences were found for alcohol or dietary behaviours (data not shown).

In planned subgroup analyses, the magnitude of the intervention effect on total physical activity was larger for those with low baseline self-rated health, however this was not statistically significant (between-group difference + 6.0 points, *p* = 0.06 vs. + 0.60, *p* = 0.07 in those with high self-rated health). A similar pattern was observed when analyses were restricted to those who had never used the Portal before (+ 4.7 points, *p* = 0.04). No between-group differences were found in any subgroup analyses for diet or alcohol intake (data not shown).

### Engagement with KT strategies

At baseline, 97.5% of participants indicated they would use email content during the intervention period compared to 70.0% for the Portal Browse page, 44.0% for Facebook, and 14.7% for Twitter (no between-group differences) (Table 3). During the intervention, 95.1% of the intervention group reported using email alerts, compared to 46.3% who browsed the Portal, 15.2% who used Facebook, and 5.3% who used Twitter. While some control group participants did report accessing content, engagement was higher in the intervention group across each strategy (Table 3). Of those who reported using each KT strategy, satisfaction (measured as perceived usefulness, and likelihood of continued use) was rated highly across all platforms (mean 5.6 to 6.5 on a 7-point Likert scale).

**Table 3:** Participant engagement and satisfaction by KT strategy

	CONT	INT	*p*
	*N* = **279**	*N* = **278**	
(At baseline) Which of the following do you plan to use to access study material:			
Email	274 (98.2)	269 (96.8)	0.41
Browse on Portal	187 (67.0)	203 (73.0)	0.15
Facebook	121 (43.4)	124 (44.6)	0.84
Twitter	35 (12.5)	47 (16.9)	0.18
	*N* = 251	*N* = 244	
Over the 12-week study period, did you access the McMaster Optimal Aging Portal via … (% Yes)			
Email	**162 (64.5)**	**232 (95.1)**	**< 0.001**
Perceived usefulness (mean ± SD)		5.91 ± 1.33	
I will continue to use (mean ± SD)		6.09 ± 1.58	
Browse on Portal	**56 (22.3)**	**113 (46.3)**	**< 0.001**
Perceived usefulness (mean ± SD)		6.10 ± 1.06	
I will continue to use (mean ± SD)		6.02 ± 1.28	
Facebook	**18 (7.2)**	**37 (15.2)**	**< 0.01**
Perceived usefulness (mean ± SD)		5.81 ± 1.27	
I will continue to use (mean ± SD)		6.16 ± 1.24	
Twitter	**3 (1.2)**	**13 (5.3)**	**0.02**
Perceived usefulness (mean ± SD)		5.62 ± 1.98	
I will continue to use (mean ± SD)		6.46 ± 1.39	
	*N* = 240	*N* = 232	
[At 3-month follow-up] Since the study ended, have you accessed the Portal via…? (% Yes)			
Email	**163 (67.9)**	**182 (78.4)**	**0.01**
Browse on Portal	92 (38.3)	103 (44.4)	0.21
Facebook	**32 (13.3)**	**51 (22.0)**	**0.02**
Twitter	**12 (5.0)**	**28 (12.1)**	**0.01**

Bold indicates statistically significant within-group difference

Qualitative data reinforced our quantitative findings, conveying that participants preferred email content over other KT strategies. They highlighted the ease of use of emails, the ability to save emails for reading later, and the ability to share information with family and friends as being the main benefits.

*…the emails, they seemed to be topic, like there was a topic and I was like okay if I'm interested in that topic I can read more. And so I liked that aspect and I liked when I clicked on something and … when it came up and it was like okay here's the main message, there's a very quick summary of something and then I can follow links if I was more interested.”*

*“It’s simple. You get it. It’s very easy to read, like it’s in point form somewhat and you see it and you go, “Oh, let’s have a look at that”.*

*“Well, so it was very convenient for me for all the obvious reasons. You can read it when you have time and you can review it and you make a file and keep your file and go back and look and reference them again, so all of those things with all the convenience of digital communication. And it was especially nice for me because I don’t choose to participate in Facebook or Twitter so it was great to be able to get the emails and also to know about the McMaster Optimal Aging Portal.”*

Qualitative data reinforced that social media was not preferred. Many participants reported not having social media accounts (particularly Twitter) and not being interested in using social media.

“*I am on social media with regard to Facebook but I haven’t - they put so much junk on that Facebook as it is I wouldn't want to, you know, you get a lot of stuff and another thing coming up on the newsfeed.”*

*"Well, I’m not on Twitter so I had no desire to join the Twitter-verse. Not that I'm anti-Twitter I'm just like, just not really that into social media. And Facebook I felt at work that was tricky, like I try not to be on Facebook at work. So I really try to limit most of my internet time to work hours and then like if I check Facebook it's really brief, like did someone message me or whatever, so really I did depend on the emails that way.”*

**Fig. 3:**
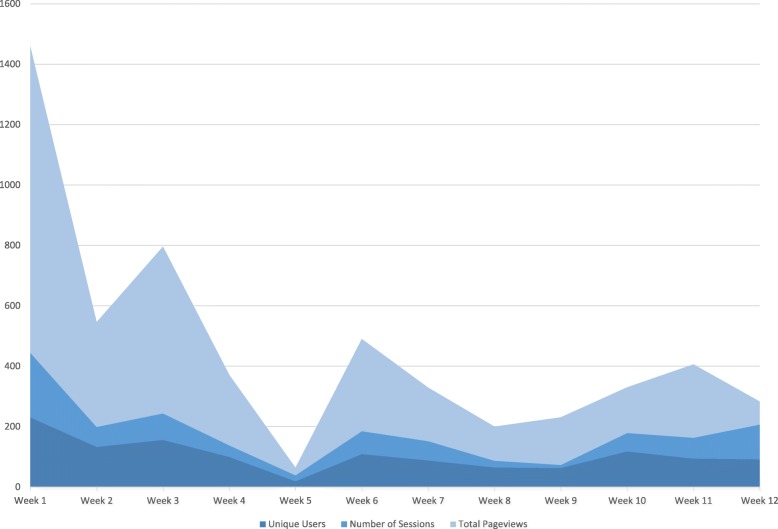
Engagement with intervention email content by week

**Fig. 4:**
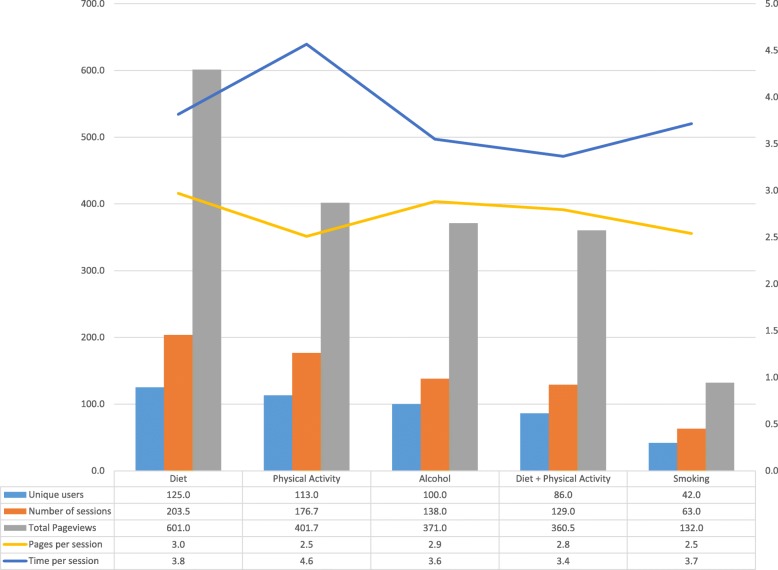
Engagement with email content by topic

Engagement with intervention content was highest during the first week of the study and lower throughout the intervention period (Fig. 3). On average, 30.1% of participants engaged with content within an email on a given week. Engagement was highest in week 1 (83.1% clicked through) and lowest in week 5 (6.8% clicked through). Data related to the number of emails received and opened were unavailable due to a technical issue with the analytics software. Number of pages per session (mean: 2.8, range 2.3 to 3.2), and time per session (mean: 4.6 min, range: 2.8 to 5.7) was consistent throughout the study period. When separated by topic (Fig. 4), engagement with intervention content related to diet and physical activity was higher than engagement with topics related to alcohol intake or smoking.

## Discussion

Based on our findings, dissemination of evidence-based information through the Portal results in small increases in knowledge of cancer prevention guidelines, and may have an impact on health behaviours, particularly in certain subgroups. Overall, we found a very small increase in the number of bouts per week of light-intensity physical activity in the intervention group at end of study, as well as a small reduction in servings per week of liquor intake at follow-up, however the small magnitude of these changes may have limited clinical significance.

Of note is the very high knowledge of cancer prevention guidelines at baseline, as well as generally positive health behaviours reported by participants, and high educational levels. Our intervention, based on the TPB, aimed to alter participants’ attitudes towards behaviour through increased knowledge and awareness of cancer risk-reduction behaviours. The likelihood of observing change was limited by the ceiling effect as a result of participants’ already high baseline knowledge.

Subgroup analyses suggest that those with lowest baseline self-rated health may experience a greater change total physical activity than those with moderate-high self-rated health at baseline, which most of our study participants were. These results replicate our team’s previous findings from the Move4Age study [47]. This study used a similar approach to deliver evidence-based information through the Portal related to physical activity and physical mobility to middle-aged and older adults. In the Move4Age study, both intervention and control group participants reported significant changes in physical activity that were maintained at the 3-month follow-up period, however there were no significant differences between groups. Interestingly, when analyses were restricted to those with low self-rated health, the intervention group reported a greater improvement in physical activity.

In both of our Portal studies to date, our study sample consisted of primarily well-educated, retired females, which is likely the result of our recruitment methods through our existing networks of Portal partners. This is consistent with findings from a systematic review of reviews, which found that the reach of interventions included in reviews of internet-delivered lifestyle behaviour change interventions was primarily limited to female, highly-educated, white individuals living in high-income countries [48]. One advantage of online compared to in-person interventions is the potential to reduce health inequities due to improved access and scalability. However, this advantage is not likely to be realized if those who may have the most to gain from an intervention (i.e., those with lower self-rated health, low socioeconomic status, rural or remote individuals with limited access to a healthcare provider) are unlikely to become engaged [49]. In an analysis from the Health Informational National Trends Survey, individuals who are older, male, or have lower education are least likely to engage in eHealth activities [50]. Further work is needed to understand how to best design, adapt and deliver interventions to underserved populations who may have the most to gain from an intervention such as the Portal.

It is well known that increasing knowledge alone is often inadequate to change behaviours to a sufficient degree that improvement in long-term health outcomes will be realized [51]. Internet-delivered interventions are often based primarily around provision of educational materials to support behaviour change in an electronic format. Recent reviews have found that incorporating additional evidence-based behaviour change techniques is important to maximize the effect of these interventions, with the number of behaviour change techniques correlated with intervention effect size [30]. Individual-tailoring, goal setting and action planning, self-monitoring, feedback, social support and social comparison, and modelling are associated with increased effectiveness of eHealth or mHealth interventions [52–54]. For example, a recent study that evaluated the effect of ‘MyPlan1.0’, a physical activity intervention for recently retired adults in Belgium, found that those who completed three online ‘modules’ which included tailored feedback that targeted intention to change for motivation, action planning and self-monitoring resulted in an increase in walking and leisure-time vigorous physical activity after one-month compared to a control group [55]. For those who design online health resources such as the Portal, it is challenging to find ways to efficiently incorporate individualization and tailoring while maintaining broad reach and generalizability. Tailoring may be accomplished in a variety of ways, either manually by a researcher (human tailoring) or expert system (computer tailoring) using developed algorithms [56]. Tailoring can range from quite simple (i.e., using the individual’s first name in materials, using a baseline assessment only) to highly complex (i.e., dynamic tailoring where ongoing monitoring or feedback informs tailoring throughout an intervention) [57]. A recent systematic review of tailored eHealth interventions targeting weight loss found a wide range of tailoring methods utilized across studies, including theoretical basis, when, how often, and how tailoring was conducted, and what variables or factors tailoring was based on [58]. Overall, the authors found that tailoring was more effective in supporting weight loss than generic interventions or wait-list controls [58]. However, in order to enhance the impact of these tools and resources, a better understanding of the components necessary for eliciting behaviour change, and specific mechanisms of tailoring that are most effective, is needed.

Another important aspect to consider when interpreting results from this and other online interventions is the actual ‘dose’ of intervention received. A previous review of 83 web-based health interventions found that only half of participants engage with the interventions in the way they were designed [59]. User engagement data collected here via Google analytics estimate that less than one-third of participants engaged with intervention content through email alerts on any given week. Our qualitative data helps to explain the apparent discrepancies between the high self-reported email use (95% reported using email during the intervention period) and low weekly usage (ranging from 6.8 to 83.1%). Participants reported one of the benefits of receiving intervention content by email was that they were able to quickly and easily self-select the content that was most interesting and relevant to them. So, while 95% reported using email, they did not do so on a weekly basis. This is not surprising given the range of topics covered related to cancer prevention. Only three individuals in our study self-identified as smokers at baseline, thus we would not expect the majority of participants to be interested in engaging in content about smoking cessation. Tailoring of intervention content by baseline behaviours, or self-reported interest (as described above) may be one way to maximize intervention engagement in future studies. Further rigorous research is needed to understand which methods of tailoring are most effective at promoting engagement, and thus having the greatest likelihood of eliciting behaviour change.

Although we were not able to track intervention group engagement with social media posts, based on self-reported usage, engagement with Twitter and Facebook content appears very low. This finding has implications for the design of future interventions in this population of older adults. Previous studies have found a positive impact of social media interventions on health behaviours such as smoking cessation [60], and weight loss [61], however these studies have been primarily conducted in younger adults whose usage patterns and preferences may be different from the older adults included in our study.

### Limitations

There are some limitations to our study that should be noted. Although we describe our study as a randomized controlled trial, given that the Portal is an already-existing publicly available website, there is the possibility that the control group accessed intervention materials related to cancer prevention during the study. While cancer prevention-related content was not promoted on the homepage, social media or regular email subscription alerts during or after the study period, we did not limit access to cancer prevention content to the intervention group only. We assessed knowledge through a series of true/false questions to identify cancer prevention guidelines. To our knowledge, there is known validated knowledge test available to evaluate these outcomes, which limits interpretability of our results. Finally, due to a technical issue we were unable to reliably estimate the number of emails received and opened. Thus it is possible that some participants in the intervention group did not receive intervention content. However, given the high proportion of intervention participants who reported receiving weekly emails, this is unlikely. Our measurement of engagement with intervention content was limited to tracking at the group level using Google analytics. It would be interesting and informative for future trials to explore whether higher engagement, or engagement with particular content was associated with a greater change in behaviour.

## Conclusion

The use of the internet and social media to disseminate information and promote behaviour change, particularly with respect to cancer prevention and treatment is growing rapidly; however evaluation of such real-world tools and websites on actual behaviour change is lacking [62]. Here we present the second in a series of studies undertaken to evaluate the impact of the McMaster Optimal Aging Portal, a freely-accessible online repository of evidence-based information on knowledge, intentions and health behaviours of middle-aged and older adults. Dissemination of evidence-based cancer prevention information through the Portal appears to improve knowledge of risk-reduction strategies, and may have a small impact on self-reported health behaviours in particular groups. There is a need to understand how best to engage with these groups, such as those with lower perceived self-rated health, who may stand to benefit most from an intervention delivered through the Portal. Next steps are to understand how tailoring of interventions and KT strategies may help to encourage more meaningful changes in health behaviours and ultimately long-term health outcomes of Portal users, and to assess whether enhanced tailoring results in greater improvements in lifestyle behaviours, as well as knowledge and attitudes in more high-needs groups.

## Data Availability

The datasets used and/or analysed during the current study are available from the corresponding author on reasonable request.

## Abbreviations

KT: Knowledge Translation
RCT: Randomized Controlled Trial
TPB: Theory of Planned Behaviour
